# Parvovirus Reactivation in COVID-19

**DOI:** 10.7759/cureus.17796

**Published:** 2021-09-07

**Authors:** Peter Y Lai, Alan Vu, Siva T Sarva, Gnananandh Jayaraman, Ramesh Kesavan

**Affiliations:** 1 Internal Medicine, Hospital Corporation of America Houston Healthcare Kingwood/University of Houston College of Medicine, Houston, USA; 2 Pulmonary and Critical Care Medicine, Hospital Corporation of America Houston Healthcare Kingwood/University of Houston College of Medicine, Houston, USA

**Keywords:** covid 19, parvovirus b-19, hypoproliferative anemia, parvovirus reactivation, immunosuppression

## Abstract

Anemia is a diagnostic challenge in patients with coronavirus disease 2019 (COVID-19). This is due to the broad differential of etiologies for anemia, which includes bleeding, bone marrow suppression secondary to sepsis, and hemolytic anemia. Here, we present a first-ever case of otherwise unexplained anemia in a patient receiving treatment for COVID-19 secondary to parvovirus B19 reactivation. While parvovirus infections often present as acute states of anemia, this patient developed a case of reactivation secondary to immunosuppression from COVID-19 treatment. This case indicates the importance of assessing for parvovirus infections in COVID-19 patients with otherwise unexplained anemia.

## Introduction

Parvovirus infections are often associated with states of profound aplastic anemia, characterized by significant decreases in hemoglobin (Hgb). While acute parvovirus infections can be seen in healthy patients, chronic parvovirus infections have been documented in various immunosuppressed conditions such as transplant recipients and acquired immunodeficiency syndrome (AIDS) [1]. Recently, the prevalence of coronavirus disease 2019 (COVID-19) has resulted in many patients developing compromised immune systems due to the use of steroids and immunomodulatory medications such as tocilizumab. Here, we document a case of otherwise unexplained anemia in a patient receiving treatment for COVID-19 secondary to reactivation of parvovirus. This case indicates the importance of assessing for parvovirus infections in COVID-19 patients with otherwise unexplained anemia.

## Case presentation

A 44-year-old female with a remote history of smoking and opiate use disorder presented with dyspnea secondary to COVID-19 pneumonia. She was diagnosed with COVID-19 at her primary care physician’s office one week prior to admission. She finished a course of steroids and antibiotics prescribed by her primary care physician but experienced worsening of her respiratory status one day prior to admission.

She was admitted to the intermediate care unit and started on high-flow nasal cannula with fraction of inspired oxygen (FiO_2_) 100% and rate of 30 L/min. She received 6 mg dexamethasone IV twice a day, enoxaparin 40 mg subcutaneously twice a day, convalescent plasma, and remdesivir. Given her hypoxic respiratory failure, she was administered tocilizumab on hospital day 2.

In the following week, the patient continued to experience worsening hypoxia. She was, therefore, treated with high doses of steroids up to 60 mg of methylprednisone IV three times a day, which was tapered down after five days. She was subsequently transferred to the intensive care unit on day 10 as she required use of bilevel positive airway pressure at 100% FiO_2_. Her Hgb was found to have decreased from 14.0 g/dL on day 12 to 10.9 g/dL on day 13. A repeat Hgb was 10.2 g/dL, and repeat Hgb checks every 6 hours showed downtrending Hgb. At this time, enoxaparin was stopped due to concern for possible retroperitoneal hematoma or gastrointestinal bleed. A computed tomography (CT) scan of the chest, abdomen, and pelvis with contrast was performed on day 14, which was negative for retroperitoneal hematoma. During this time, no melena, hematochezia, or any other signs of bleeding were noted. The patient was on empiric piperacillin/tazobactam, azithromycin, voriconazole, and acyclovir at that time, with no evidence to suggest a superimposed infection.

A workup for hemolytic anemia was subsequently initiated and hematology was consulted on day 14. A peripheral smear showed no significant hemolysis and haptoglobin was 355 mg/dL (NL 42-296). A direct Coombs test and HIV were negative. Reticulocyte count was 1.9% with nucleated erythrocytes present (reticulocyte index of 0.52), consistent with reduced erythrocyte production. Given the otherwise negative workup, the patient was evaluated for other causes of hypoproliferative anemia including parvovirus. Parvovirus immunoglobulin G (IgG) was positive while immunoglobulin M (IgM) was negative. On hospital day 16, the patient’s Hgb decreased to a nadir of 6.8, which required a transfusion of 1 unit of packed red blood cells. Since the patient was becoming increasingly anemic and increasingly dyspneic, 25 g of intravenous immunoglobulin (IVIG) was administered. A polymerase chain reaction (PCR) assay for parvovirus was positive on day 17. By day 22, the patient’s Hgb had increased to 10.4, with a reticulocyte count of up to 4.6% with nucleated erythrocytes present (reticulocyte index 1.21). After her anemia had improved, she did report significant symptomatic improvement in her dyspnea. The patient eventually improved over the next two weeks and was eventually discharged.

## Discussion

In patients with COVID-19 infections, a broad differential exists for anemia. As many COVID-19 patients receive therapeutic anticoagulation, bleeding must be excluded as a cause of anemia [2]. Furthermore, superimposed infections resulting in sepsis should also be considered in patients. Finally, severe cases of autoimmune hemolytic anemia have been documented in patients with COVID-19 [3]. However, a literature search of PubMed with the keywords COVID and hypoproliferative anemia showed no prior documented cases. For our patient with hypoproliferative anemia, a workup was eventually suggestive of a parvovirus reactivation.

Parvovirus B19 is a common pathogen in humans. Most infections are self-limited due to an appropriate immunological response to the virus. Thus, parvovirus reactivation and subsequent aplastic anemia is commonly associated with immunocompromised patients such as transplant or AIDS patients. The pathophysiology behind the anemia is likely related to parvovirus’ predilection for erythrocyte progenitor P antigens, inhibiting erythrocyte development [4]. Therefore, treatment for parvovirus-related anemia commonly involves correcting the immunosuppression or utilizing IVIG to neutralize parvovirus antigen binding [5].

For our patient, we suspect her anemia was due to an acute reactivation of parvovirus. Reactivation infections have been documented in transplant recipients and patients with AIDS [1]. Because our patient was severely immunocompromised from COVID-19 treatment, which included high-dose steroids and tocilizumab, she was at high risk for reactivation. The patient’s negative IgM, positive IgG, and positive PCR levels were consistent with parvovirus reactivation [1]. The negative IgM is due to the patient’s profound immunosuppression. The positive IgG is indicative of prior infection. Finally, the positive PCR is characteristic of ongoing infection, consistent with reactivation. In this particular case, treatment with IVIG led to significant improvement in the patient’s anemia and reticulocyte counts, similar to other cases of parvovirus reactivation.

## Conclusions

A broad differential exists for anemia in patients with COVID-19 infections. A thorough workup must be initiated, which includes possible sources of bleeding, hemolytic anemia, and superimposed infections. However, in patients with negative workups for these more common causes, it is important to consider parvovirus infections, particularly in patients with hypoproliferative anemias. Patients undergoing active treatment for COVID-19 are significantly immunosuppressed, which may result in parvovirus reactivation as in this patient. Notably, IgM may be erroneously negative in these patients due to immunosuppression; hence, checking parvovirus PCR levels is important for diagnosis.
